# Experimental Evidence of Biological Interactions among Different Isolates of *Trypanosoma cruzi* from the Chaco Region

**DOI:** 10.1371/journal.pone.0119866

**Published:** 2015-03-19

**Authors:** Paula G. Ragone, Cecilia Pérez Brandán, Mercedes Monje Rumi, Nicolás Tomasini, Juan J. Lauthier, Rubén O. Cimino, Alejandro Uncos, Federico Ramos, Anahí M. Alberti D´Amato, Miguel A. Basombrío, Patricio Diosque

**Affiliations:** 1 Unidad de Epidemiología Molecular, Instituto de Patología Experimental, CONICET (Consejo Nacional de Investigaciones Científicas y Técnicas), Universidad Nacional de Salta, Salta-Capital, Argentina; 2 Instituto de Patología Experimental, CONICET (Consejo Nacional de Investigaciones Científicas y Técnicas), Universidad Nacional de Salta, Salta-Capital, Argentina; 3 Cátedra de Química Biológica, Facultad de Ciencias Naturales, Universidad Nacional de Salta, Salta-Capital, Argentina; Albert Einstein College of Medicine, UNITED STATES

## Abstract

Many infectious diseases arise from co-infections or re-infections with more than one genotype of the same pathogen. These mixed infections could alter host fitness, the severity of symptoms, success in pathogen transmission and the epidemiology of the disease. *Trypanosoma cruzi*, the etiological agent of Chagas disease, exhibits a high biological variability often correlated with its genetic diversity. Here, we developed an experimental approach in order to evaluate biological interaction between three *T*. *cruzi* isolates belonging to different Discrete Typing Units (DTUs TcIII, TcV and TcVI). These isolates were obtained from a restricted geographical area in the Chaco Region. Different mixed infections involving combinations of two isolates (TcIII + TcV, TcIII + TcVI and TcV + TcVI) were studied in a mouse model. The parameters evaluated were number of parasites circulating in peripheral blood, histopathology and genetic characterization of each DTU in different tissues by DNA hybridization probes. We found a predominance of TcVI isolate in blood and tissues respect to TcIII and TcV; and a decrease of the inflammatory response in heart when the damage of mice infected with TcVI and TcIII + TcVI mixture were compared. In addition, simultaneous presence of two isolates in the same tissue was not detected. Our results show that biological interactions between isolates with different biological behaviors lead to changes in their biological properties. The occurrence of interactions among different genotypes of *T*. *cruzi* observed in our mouse model suggests that these phenomena could also occur in natural cycles in the Chaco Region.

## Background

Advances in molecular typing techniques show that many infectious diseases may arise from co-infections or re-infections with more than one genotype of the same pathogen. In these mixed infections the co-infecting parasites may be interacting among each other within the same host determining host fitness, severity of disease symptoms, parasite transmission successful rate and epidemiology of the disease [1]. Various mechanisms can cause interactions between parasite species or among different genotypes of the same species within an individual host. For example, parasites can infect the same target site within a host and directly interact among each other by interference competition, or indirectly by resources competition or via the host immune system [2].

In general, biological interactions between protozoan parasites have been divided into two main groups: those who involved parasites belonging to the same species and the ones that occur between closely to distantly related different species [3]. In this sense, several research studies have reported mixed infections in *Leishmania spp* [4,5,6], *Plasmodium spp* [7,8,9], *Trypanosoma brucei* and *Trypanosoma congolense* [10,11,12].

*Trypanosoma cruzi* is the etiologic agent of Chagas disease, an illness that affects several million people in Latin America and still remains an important public health problem in certain endemic areas of Argentina. This parasite shows a high genetic variability which has been the basis to classify it into six Discrete Typing Units (DTUs), TcI to TcVI [13]. In addition to this genetic diversity, *in vitro* and *in vivo T*. *cruzi* infection models showed a high biological variability among different genotypes of *T*. *cruzi* [14,15,16,17,18,19]. Although it is supposed that genetic and biological diversities of the parasite are essential to determine the clinical course of Chagas disease, specific associations between particular clinical manifestations and a determined lineage have not been clearly demonstrated [20]. Furthermore, the host genetics and its ability to establish an immune response to control the infection are very important in the outcome of the disease [21].

The consequences of mixed infections by different *T*. *cruzi* DTUs have been studied in animal models using different laboratory strains. It has been demonstrated *in vivo* that the tissue tropism of one *T*. *cruzi* genotype could change in the presence of another genotype of a different DTU [22,23]. Even more, the histopathological damage and the intensity of the inflammatory process resulting of these co-infections also present remarkable variations [24,25]. Other studies involving *T*. *cruzi* mixed infections showed that the parasite load in peripheral blood could be altered either increasing or decreasing according to the co-infecting strains [26,27,28]. Even the outcome of specific chemotherapy has been proven to be altered by these events of concomitant infection by *T*. *cruzi* [26,29].

In several geographical areas of the Southern Cone of America, the occurrence of natural mixed infections by different genotypes of *T*. *cruzi* have been widely reported in humans [20,30,31,32,33], in wild and domestic animals [34,35] and in the vector *Triatoma infestans* [34,36].

In a previous work we described the different biological properties displayed by three selected isolates obtained from the Chaco region of Argentina and belonging to DTUs TcIII, TcV and TcVI [17]. These isolates have the particularity of circulating sympatrically in a restricted geographical area; therefore, here we describe the biological outcome resulting of *in vivo* experimental dual-mixed infections with these *T*. *cruzi* strains. Our working hypothesis is the existence of biological interactions among different *T*. *cruzi* isolates in the vertebrate host.

## Methods

### Ethics statement

All animal protocols adhered to the National Institutes of Health (NIH) ‘‘Guide for the care and use of laboratory animals” and were approved by the Animal Ethics Committee of the School of Health Sciences, National University of Salta (Nu 014–2011) [37].

### *Trypanosoma cruzi* isolates

Different *Trypanosoma cruzi* isolates were examined in the present work. These isolates were obtained from Las Leonas settlement (W 61° 39’ 8.7”, S 27° 01’ 49”), located in the South-west of Chaco Province, Argentina. The protocol of obtaining samples was approved by the Bioethics Committee of the Faculty of Health Sciences of the National University of Salta, Argentina (Resolution N°052–10). The inhabitants signed an informed consent form before sampling at each house. This study did not involve endangered or protected species.

Parasites were recovered from the feces of either, naturally infected *Triatoma infestans* or insects used for xenodiagnosis of mammalian hosts. The isolates were identified as DTUs TcIII (LL051-P24), TcV (LL014–1) and TcVI (LL040–1) by Multilocus Sequence Typing (MLST) technique, using a typing scheme proposed by Lauthier and cols, [38]. These parasites were maintained in a vector transmission model developed by Ragone and cols, (2012). Hereafter, each isolates will be named according to the corresponding DTU.

### Experimental infection in mice

Six groups of 4 male C57BL/6J mice (one month old) were inoculated by intraperitoneal (i.p.) route with parasites recovered from the feces of infected insects and each infected group was followed during 30 days after infection. Prior infection, the feces were visualized microscopically, in order to distinguish epimastigotes, metacyclic and intermediate forms (parasites whose morphology is intermediate between epimastigotes and tripomastigotes); according to Kollien and cols. [39]. The final inoculation dose was adjusted according to the amount of metacyclic and intermediate forms. For single infections with TcIII, TcV or TcVI, 10^4^ parasites were inoculated per mouse. Instead, 5x10^3^ parasites from each isolate were inoculated for dual mixed infections (TcIII + TcV, TcIII + TcVI and TcV + TcVI). We decided to maintain constant the final dose used in both, single and dual-mixed infections, in order to avoid possible differences due to the final number of inoculated parasites. For this reason, we examined in previous assays if the two doses, 10^4^ and 5x10^3^ parasite/mouse of each isolate show differences in the biological parameters to be studied. No statistical differences were observed between these doses (data not shown). Control groups inoculated with Phosphate Buffer Saline (PBS) were included in the experiment. Animal care guidelines adopted by the Health Sciences Faculty, National University of Salta, Argentina, were strictly followed.

### Biological parameters evaluated

#### Parasitemia

Fresh blood from inoculated animals was collected in heparinized glass capillary pipettes by sectioning the tail tip under slight anesthesia. Ten microliters (μl) of blood were placed between slide and cover slip and the number of parasites per 100 fields was recorded microscopically (400X) at different time points.

#### Histopathology

Animals were sacrificed at 30 days post infection (dpi) by exposure to halothane, and cardiac and skeletal muscle samples were collected. Tissue samples were divided into two parts; one was stored at -80°C for DNA extractions and the other part was fixed in 10% formalin and processed using routine histological techniques. Serial histological sections (3 to 5 μm thick), were stained with hematoxylin-eosin and observed under microscope (50, 200 and 400X). Quantification of the inflammatory response (IR) was assessed taking into account the presence and intensity of inflammatory foci. Criteria were set according to the size and number of foci in order to quantify the inflammatory process in different organs. Thus, IR was blindly quantified as null, mild, moderate and severe as reported in Ragone and cols. [17]. In some cases intermediate values for the inflammatory response were admitted: mild to moderate and moderate to severe.

### Detection of *T*. *cruzi* DNA in blood and tissue samples

Peripheral blood was obtained at 30 dpi from each animal (350 μl) and mixed with 700 μl of guanidine buffer. DNA extractions were performed from 100μl of the mixture blood-guanidine buffer by using the phenol-chloroform method. DNA extractions from a skeletal and cardiac muscle sample (obtained at 30 dpi) were performed using a kit (PureLink Genomic DNA Kit, Invitrogen). Subsequently, PCR amplification of 330 bp corresponding to minicircle hypervariable regions (mHVR) was performed using primers 121 (5’-AATAATGTACGGG(T/G)GAGATGCATGA-3’) and 122 (5’-GGTTCGATTGGGGTTGGTGTAATATA-3’) [40]. Amplification was performed in a MJR PTC-100 thermocycler (MJ Research, Watertown, MA, USA). The reaction products were visualized in a 2% agarose gel stained with ethidium bromide.

### Identification of *T*. *cruzi* Discrete Typing Units in biological samples

Detection of each isolate was carried out by hybridization with specific mHVR-kDNA non-radioactive probes in blood and tissues samples taken at 30 dpi. The probes were constructed using DNA from isolates LL051-P24 (TcIII), LL055R3cl2 (TcV) and CL-Brener (TcVI). The primers for probe construction were CV1 (5’-GATTGGGGTTGGAGTACTAT-3’) and CV2 (5’-TTGAACGGCCCTCCGAAAAC-3’) which produced a 290-bp fragment. Restriction sites for Sau96I and ScaI which allow elimination of the minicircle constant region of these PCR fragments were included in the sequence of these primers [41]. PCR fragments were further digested with the restriction endonucleases obtaining a 250-bp final product. The specificity of each generated DNA probe was evaluated in our laboratory by Southern blot analysis against different 330-bp mHVR PCR products of several *T*. *cruzi* isolates belonging to the different DTUs [42]. Briefly, Southern blot analysis was performed using 10 μl of each 121–122 PCR product. Samples were subjected to electrophoresis, transferred to Hybond N + nylon membranes (Roche Diagnostics) and cross-linked with UV light to fix the DNA. The membranes were pre-hybridized for at least 30 minute at 42°C and individually hybridized with the generated probes. Labeling of the probe and DNA hybridization were performed according to the protocol supplied with the PCR-DIG DNA-labeling and detection kit (Roche Applied Science, USA).

For assessing the limit of detection of the technique, *T*. *cruzi* DNA of the different isolates involved in the study was analyzed individually and combined. The limit of the technique in detecting single infections was 0.1 fg/μl for TcIII and TcV DTUs, and 0.5 fg/μl for TcVI DTU. Whereas that in the mixed infections we evaluated different proportions of one isolate combined with other isolate, from 0.1 fg/μl to 0.9 fg/μl. As a result of this experiment, the detection limit for TcV was 0.1 fg/μl in the TcIII + TcV and TcV + TcVI mixtures; while the detection limit for TcIII was 0.5 fg/μl in TcIII + TcV and TcIII+TcVI. Finally the detection limit for TcVI in the mixtures TcV+TcVI and TcIII+TcVI was 0.5 fg/μl.

### Statistical analysis

Differences in the number of parasites in peripheral blood and inflammatory response among groups were evaluated using one-way variance analysis (ANOVA). To analyze inflammatory response, numeric values for the different levels were assigned: null: 0, mild: 1, mild to moderate: 2, moderate: 3, moderate to severe: 4, and severe: 5. Statistical analysis was performed using the software GraphPad Prism V5.00.

## Results

### Predominance of the more virulent isolate in peripheral blood and DTU detection by mHVR-kDNA hybridization

**Fig 1:**
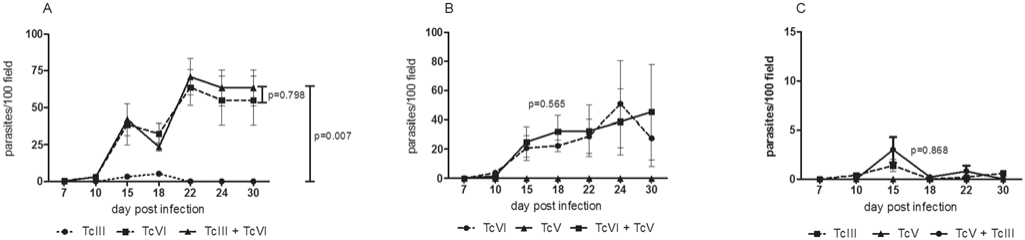
Parasitemia in peripheral blood of singles and mixed infections. This variable was measured by microscopic observation, of animals inoculated with single and mixed isolates. (A) Shows difference between TcIII vs TcVI vs TcIII + TcVI, (B) TcVI vs TcV + TcVI and (C) TcIII vs TcIII + TcV. The parasitemia of TcV isolate was sub-patent.

As in a previous study Ragone and cols. [17] in this work a marked difference was well established respecting the parasitemia between the three different isolates. The TcV isolate presented non-detectable parasitemia in fresh blood mounts. However, PCR assays corroborated infection by this isolate. On the other hand, circulating parasites in peripheral blood were detected in mice inoculated with TcIII and TcVI, being the parasitemia of TcVI significantly higher than the one obtained by TcIII (p = 0,013). When co-infection models (TcIII + TcVI, TcIII + TcV and TcV + TcVI) were considered, the pattern of parasitemia was the one corresponding to the more virulent DTU in all cases (Figs. 1A-C). In the co-infection involving TcIII + TcVI and TcV + TcVI, the parasitemia was the one described for TcVI alone. In addition, in the mixture TcIII + TcV, a behavior equal to TcIII was observed. As expected, there were non-significant differences between TcVI vs. TcIII + TcVI and TcVI vs. TcV + TcVI; neither between TcIII vs. TcIII + TcV. No statistical comparison between TcV vs. TcIII + TcV or TcV + TcVI was carried out since no circulating parasites were detected for TcV isolate.

**Fig 2:**
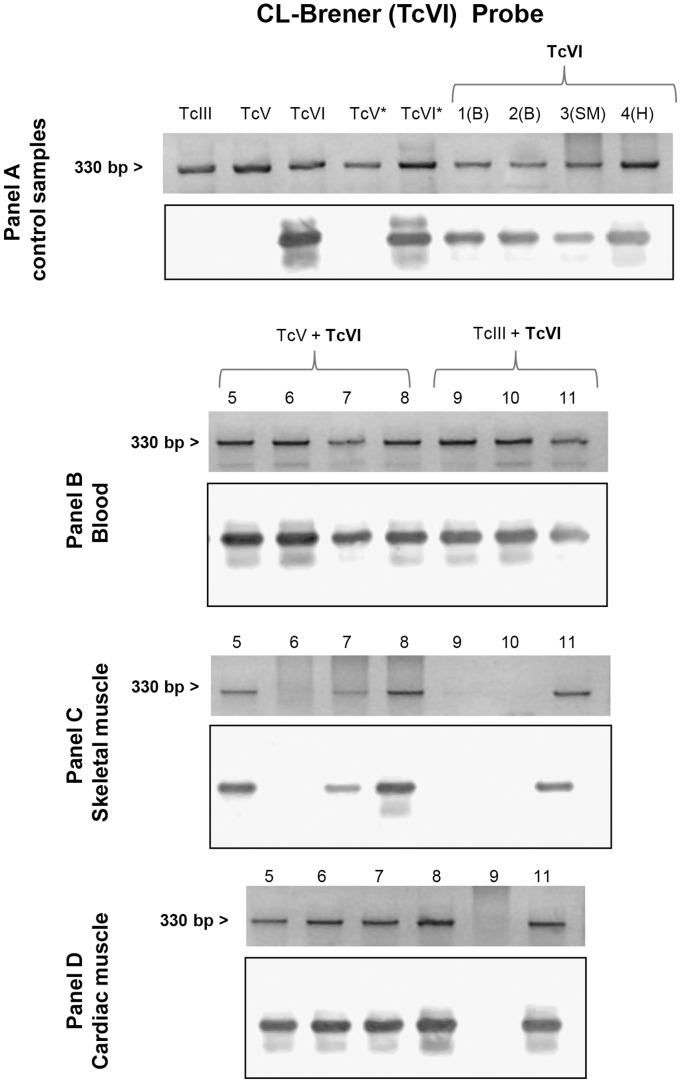
Southern blot analyses using the TcVI (CL-Brener) probe. Each panel shows the electrophoretic pattern of minicircle regions and kDNA transferred to a nylon membrane. Panel A, lanes TcIII, TcV, TcVI, TcV* and TcVI* correspond to DNA of parasite culture from LL051-P24 (DTU TcIII), LL055R3cl2 (DTU TcV), CL-Brener (DTU TcVI), LL014–1 (DTU TcV*) and LL040–1 (DTU TcVI*) respectively; lane 1–4: blood (B), skeletal muscle (SM) and heart (H) samples of mouse infected with TcVI isolate. The asterisk as superscript of the DTU indicates DNA sample from culture of the same inoculated isolate. Panel B, C and D: blood, skeletal muscle and cardiac muscle, respectively, of animals infected with TcV + TcVI (Lane 5–8) and TcIII + TcVI (Lane 9–11).

**Fig 3:**
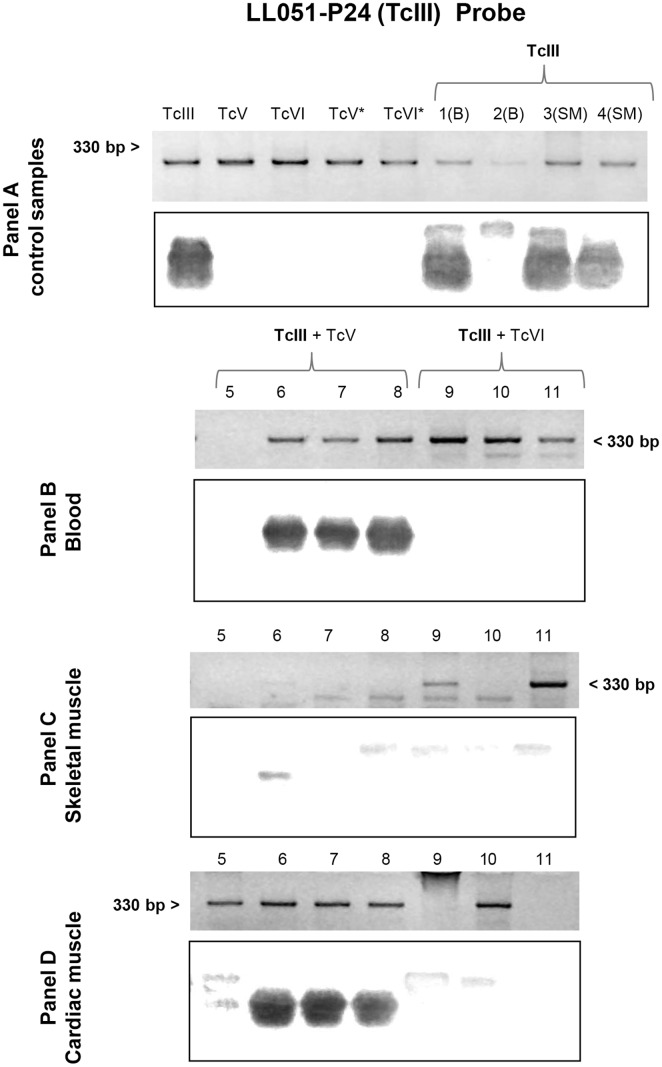
Southern blot analyses using the TcIII (LL051-P24) probe. Each panel shows the electrophoretic pattern of minicircle regions and kDNA transferred to a nylon membrane. Panel A: lane TcIII, TcV, TcVI, TcV* and TcVI* correspond to DNA of parasite culture from LL051-P24 (DTU TcIII), LL055R3cl2 (DTU TcV), CL-Brener (DTU TcVI), LL014–1 (DTU TcV*) and LL040–1 (DTU TcVI*) respectively; and lane 1–4: blood (B) and skeletal muscle (SM) samples of mouse infected with TcIII isolate. The asterisk as superscript of the DTU indicates DNA sample from culture of the same inoculated isolate. Panel B, C and D: blood, skeletal muscle and cardiac muscle, respectively, of animals infected with TcIII + TcV (Lane 5–8) and TcIII + TcVI (Lane 9–11).

**Fig 4:**
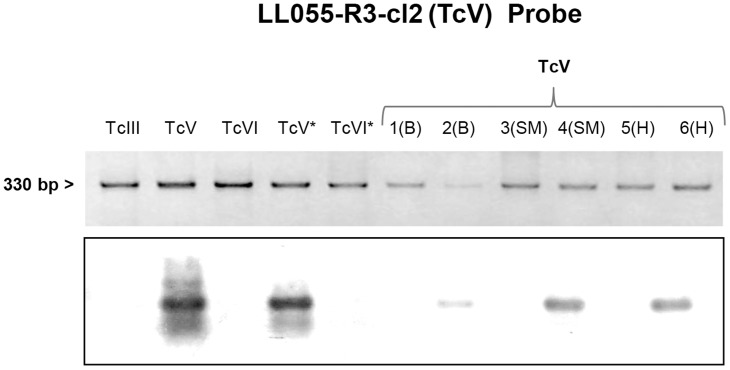
Southern blot analyses using the TcV (LL055R3cl2) probe. Electrophoretic pattern of minicircle regions and kDNA transferred to a nylon membrane. Lane TcIII, TcV, TcVI, TcV* and TcVI* correspond to DNA of parasite culture from LL051-P24 (DTU TcIII), LL055R3cl2 (DTU TcV), CL-Brener (DTU TcVI), LL014–1 (DTU TcV) and LL040–1 (DTU TcVI), respectively; and lane: 1–6 blood (B) skeletal muscle (SM) and cardiac muscle (H) of mouse infected with TcV isolate.

Finally, in blood samples collected at 30 days post-infection we applied specific mHVR-kDNA hybridizations to determine the circulating DTU in single and dual infections. TcVI was identified in all the infection formulas involved (TcIII + TcVI and TcV + TcVI mixtures) (Fig. 2B), while TcIII was only detected in the mixture TcIII + TcV (Fig. 3B). Even though no visible parasites were detected for TcV, positive PCR could be obtained (Fig. 4); however, no hybridization signal for TcV was detected in the mixture TcV + TcVI. In addition, for the blend TcV + TcIII, only TcIII could be detected (Fig. 3B). Table 1 shows the number of mice with positive signal for each specific probe.

**Table 1:** mHVR-kDNA hybridization results for the detection of individual DTUs in mixed infections.

MIXTURES	SAMPLES	Probe TcIII	Probe TcV	Probe TcVI
**TcIII + TcV**	Blood	3/4	0/4	-
	Skeletal muscle	1/4	0/4	-
	Heart	4/4	0/4	-
**TcIII + TcVI**	Blood	0/3*	-	3/3
	Skeletal muscle	0/3*	-	2/3*
	Heart	0/2*	-	1/2*
**TcV + TcVI**	Blood	-	0/4	4/4
	Skeletal muscle	-	0/4	3/4
	Heart	-	0/4	4/4

The values correspond to mice with positive hybridization for a specific probe in relation to the total of mice inoculated with the mixture.

* indicate one or two sample lost.

### Histopathological damage and its association with the different infecting DTUs

When single infections were considered, different patterns of tissue damage were observed. TcVI isolate induced significantly more histological lesions in skeletal muscle (p = 0.0026) and heart (p<0.0001) than TcIII and TcV. In addition, TcIII produced significantly more damage in heart than TcV (p<0.0001). Briefly, TcVI induced severe damage in heart and moderate damage in skeletal muscle; additionally amastigote nests and fiber homogenizations were found in both tissues in animals infected with this isolate. On the other hand, TcIII induced mild-moderate lesions in heart and skeletal muscle and no amastigote nests or cellular alteration were found; while TcV induced only mild lesions in the analyzed tissues.

However, in our co-infecting models a moderate damage in heart and skeletal muscle was found in the mixtures TcIII + TcVI and TcV + TcVI, while in mice infected with TcIII + TcV, mild histological lesions were observed in both tissues. In addition, amastigote nests were found in heart and skeletal muscle of animals infected with TcVI + (TcIII or TcV).

**Fig 5:**
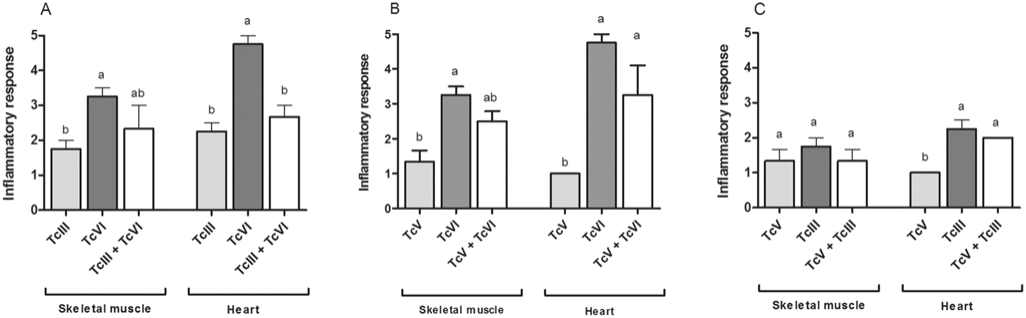
Histological damage in mice infected with single and mixed infections. Inflammatory response observed in skeletal muscle and heart samples of experimental groups inoculated with: (A) TcIII, TcVI or TcIII + TcVI, (B) TcV, TcVI or TcV + TcVI and (C) TcIII, TcV or TcIII + TcV. Different letters above error bars indicate statistical difference (p<0.05).

In cardiac muscle, no differences were detected between the damage induced by TcIII or by TcIII + TcVI co-infection; however, the intensity of the lesions induced by this mixture were significantly milder respect to the damage induced by TcVI (p = 0,0003) (Fig. 5A). Instead, the histological lesions in heart of animals infected with TcV + TcVI were statistically different from the ones detected in animals infected with TcV alone (p = 0.002) but not to the produced by TcVI (Fig. 5B). The same results were observed when the damage in heart of mice infected with TcIII + TcV was compared to the one of mice infected with TcIII; in this case, no statistical differences between single and mixed infections were detected; however, the lesions induce by the mixture were significantly different than the induce by TcV alone (p = 0,001) (Fig. 5C).

In skeletal muscle, the intensity of the lesions found in mice infected with TcIII + TcVI and TcV + TcVI mixtures was intermediate respect to each single infection; however, no statistical differences were detected (Fig. 5A and 5B). In TcIII + TcV co-infection the damage in skeletal muscle was equal to the one detected in single infections with TcIII or TcV (Fig. 5C).

Heart and muscle tissues were analyzed by PCR and posterior DTU specific hybridization assays. *T*. *cruzi* DNA was detected in both, skeletal and cardiac muscle, in all experimental groups. In addition, we found the presence of the isolates TcVI or TcIII in the mixed infections (Fig. 2C and D and Fig. 3C and D). Again, although TcV probe worked correctly (Fig. 4), no hybridization against positive PCR samples of animals infected with TcV + TcIII or TcV + TcVI mixtures was observed. DTU detection by hybridization tests is summarized in Table 1.

## Discussion

Here, we developed an experimental approach in order to evaluate if mixed infections involving *T*. *cruzi* isolates, belonging to different DTUs and collected from a restricted area in the Chaco Region of Argentina, display evidence of biological interaction in a mouse model. In this work we analyzed the parasitemia and histological damage in heart and skeletal muscle of C57BL/6J mice infected with a combination of the different isolates analyzed. The intrinsic properties of the isolates under study were as follow: TcVI isolate induces high parasitemia, severe histological damage in heart and moderate lesions in skeletal muscle. On the other hand, the TcIII isolate induces less parasitemia than TcVI, and mild-moderate histological damage in heart and skeletal muscle. Finally TcV isolate induces sub-patent parasitemia and mild lesions in the analyzed tissues. When TcVI was combined, either with TcIII or TcV, only the parasitemia pattern of TcVI was observed, suggesting that TcVI isolate predominates over TcIII and TcV isolates. This result was supported by the fact that only DNA of TcVI was detected in the co-infection models involving this isolate and TcIII or TcV. Similar results were obtained for the mixture TcIII + TcV, where the observed parasitemia pattern corresponded to the TcIII pattern and only DNA of TcIII was detected by the probes. This result could be due to several factors: the survival of one isolate in peripheral blood could be related to different mechanisms associated with its ability to escape from the host immune system [28], or due to a selective process within the host cells in favor of a given DTU [43]. On the other hand, in a previous work we reported that the isolates TcIII and TcVI induce a higher serological response than TcV [17]; in consequence we believe that in a mixed infection event between TcV + (TcIII or TcVI), the isolate TcV may be susceptible to the immunological response induced by TcIII or TcVI. Therefore, TcV availability in the host would be reduced, or even eliminated from the host, and not detected at least by the technique herein applied.

When cardiac muscle samples of mice infected with the mixtures TcIII + TcVI and TcV + TcVI were analyzed; only presence of TcVI isolate was confirmed by hybridizations assays. However, cardiac lesions induced by TcIII + TcVI mixture were the same that the induced by TcIII isolate alone, suggesting that the presence of TcIII, perhaps in an early infection, modifies the alterations that TcVI alone could cause in the co-infected mice, in spite of the TcIII isolate is not detected in this mixture. On the other hand, skeletal muscle samples of animals co-infected with TcVI + (TcV or TcIII) showed intermediate values of damage compared to single infections, indicating that the combination of two isolates could modify the expected lesions in this tissue. These results could be due to the ability of each isolate to infect cells since it is known that *T*. *cruzi* is capable of infecting a wide variety of host cells, but the persistence of this parasite in cardiac and skeletal muscles depends on its ability to enter in the host cell and in the interaction with this [44]. On the other hand, it has been reported that the co-infections between different strains of *T*. *cruzi* trigger both protective inflammatory immunity and regulatory immune mechanisms that attenuate the damage in heart in a mouse model [25].

Based on these observations we evidence biological interaction in our murine co-infection model. However, it is important to note that the prevalence of either one isolate or the another could vary according to the infection period in which blood samples are taken [25,28,45] as well as the analyzed tissue [22].

In spite of several studies demonstrating the presence of biological interactions within mammalian hosts [23,25,27,28,46], these works analyzed strains of *T*. *cruzi* from very different geographical regions. In the present work, the three *T*. *cruzi* isolates studied were obtained in the same temporal period, in the same geographical area and simultaneously introduced into the animal model.

Is important to note that the DTUs studied in this work have epidemiological relevance, since the TcV and TcVI are more prevalent in domestic cycles [34,42,47,48], whereas the TcIII DTU was found in domestic animals [34]. It is clear that the extrapolation of co-infection results obtained from animal models to the natural occurrence of the phenomena in other hosts should be carefully considered. We emphasize the predominance of TcVI in the mixture TcV + TcVI since this mixture was found in human blood samples in endemics areas of Argentina [42,49].

Many studies have shown that the biological differences between closely related DTUs are smaller than the biological differences among genetically divergent DTUs [14,15,19,27]. However, an interesting question that emerges from this work is why two genetically close strains of *T*. *cruzi*, as TcV and TcVI, exhibit opposite biological properties. Even the biological interaction between them could lead to the predominance of one isolate (TcVI) over the other (TcV), at least during the acute phase and in our specific experimental model. In this sense we believe that the interaction with the host immune response, as well as the mechanisms related to the regulation of the acute inflammatory response and the proteomic expression of different DTUs could also be contributing to the pathology of each isolate and its interactions.

## Conclusions

The results presented in this work show that biological interactions among different genotypes of *T*. *cruzi* from the Chaco Region do occur in our experimental model. Consequently, our results demonstrate that the biological interaction between *T*. *cruzi* strains in a mammal host is phenomenon that could be occurring in natural cycles. However, to examine this hypothesis field studies involving natural hosts should be performed.
